# miR-31 and miR-145 as Potential Non-Invasive Regulatory Biomarkers in Patients with Endometriosis

**DOI:** 10.22074/cellj.2018.4915

**Published:** 2018-01-01

**Authors:** Oranous Bashti, Mehrdad Noruzinia, Masoud Garshasbi, Morteza Abtahi

**Affiliations:** 1 Department of Medical Genetics, Faculty of Medical Sciences, Tarbiat Modares University, Tehran, Iran; 2 Dena Hospital, Shiraz, Iran

**Keywords:** Biomarker, Endometriosis, miR-145, miR-31, miRNA

## Abstract

**Objective:**

Endometriosis is a prevalent gynecologic disease affecting 10% of women in reproductive age. Endometriosis is diagnosed by laparoscopy that was followed by histologic confirmation. Early diagnosis will lead to a more effective treatment with much less morbidity. As miR-31 and miR-145 are shown to be directly or indirectly correlated to biological processes involved in endometriosis, the aim of this study was to examine the association of miR-31 and miR-145 expression in plasma with the presence of endometriosis.

**Materials and Methods:**

In this case control study, the plasma samples of 55 patients with endometriosis and 23 women without endometriosis were collected, extracted and analyzed by real time quantitative polymerase chain reaction (qPCR) for the expression of miR-145 and miR-31.

**Results:**

Our findings showed that miR-31 expression levels in stage 3 or 4 and stage 1 or 2 were significantly down- regulated (less than 0.01-fold, P<0.05), while the expression level of miR-145 was significantly up-regulated in women with endometriosis in stage 1 or 2.

**Conclusion:**

Different cellular biological processes, such as differentiation, proliferation, mitochondrial function, reactive oxygen species (ROS) production, invasion and decidualization, are deregulated in endometriosis. miR-31 and miR-145 are microRNAs (miRNAs) with potential roles, as shown in pathologies like cancers. We found that miR- 31 was under-expressed in patients with endometriosis, while miR-145 was over-expressed in stage 1 or 2, indicating that they were relatively down-regulated in the more severe forms. Our findings suggested that these two miRNAs may be considered as potential biomarkers with probable implications in early diagnosis and even follow-up of patients with endometriosis.

## Introduction

Endometriosis is an endometrial inflammatory disease that affects 5 to 10% of women in reproductive age. Its main feature is the presence of endometrium-like tissue in sites other than the uterus. These extra uterine tissues appear mostly on the ovaries and peritoneum. The main clinical symptoms are chronic pelvic pain, dyspareunia, and infertility. Although the exact mechanism and pathology of the disease remains unclear, there are distinct molecular and immunologic differences between normal endometrium and eutopic or ectopic endometriosis tissue. Overproduction of estrogen and cytokines, progesterone resistance, and most importantly epigenetic deregulation of gene expression in endometriotic tissue are recently investigated. Gene-expression profiling of endometrium from women with endometriosis has revealed the candidate genes related to implantation failure, infertility, and progesterone resistance as compared to endometriosisfree women (1, 2).

Several studies have failed to show a major pure genetic contribution in endometriosis. However, a number of studies have emphasized on epigenetics as a major underlying pathogenic mechanism. For example, genome-wide methylation analysis has indicated that in endometriosis, there is DNA methylation abnormalities, like expression of clusters of genes involved in differentiation through GATA family transcription factors (3), which are the proteins that bind to DNA and regulate many functions, like differentiation. Progesterone resistance and abnormal decidualization present in endometrial tissues are the other supporting evidences for differentiation defect in endometriosis (4). These findings have indicated that epigenetic deregulation might be involved in the main biological processes found in endometriosis (5).

Endometriosis is also known as a stem cell disorder that is the underlying mechanism in extra endometrium implantation. Although the exact stem cell pathology and its mechanisms are still unclear, abnormalities in cell motility and invasive capacity have been reported in endometriosis (6). Furthermore, it has been shown that decreased level of proteins expression involved in cell adhesion and cytoskeleton and lower level of proteolytic activity promote endometriotic lesion growth in endometriosis (7-11). These abnormalities in stem cells might be in part mediated by microRNAs (miRNAs) deregulations. It has been mentioned that an abnormal decrease or increase in miRNAs expression in endometriotic stromal cells lead to up-regulation of miR-503 expression, down-regulation of microRNA, inhibition of proliferation, as well as promotion of apoptosis (12). However, proliferation of endometrial stromal cells (ESC), cell invasiveness and motility are known to be increased in endometriosis, mostly by epigenetic mechanisms and miRNAs (13).

miRNAs are non-coding RNAs of 18-25 nucleotide long that regulate post-transcriptional gene expression by hybridizing to the complementary regions of target mRNA, leading to inhibition of translation with or without degradation of the mRNA level. Among miRNAs, miR31 is a key player in spermatogenesis, implantation, and embryo development (14). Furthermore, miR-31 controls several vital processes. In breast cancer and liver cancer, down-regulated miR-31 acts as a tumor suppressor, while in colorectal cancer, cervical cancer and lung cancer, up- regulated miR-31 is considered as oncomir. In addition, miR-31 acts as a prognostic biomarker. High expression level of miR-31 is correlated to shorter survival in patients with malignant pleural mesothelioma, whereas normal/ low expression of miR-31 is associated with longer survival in this patients (15). miR-31 also plays important roles in fertility and pathogenesis of endometriosis through down-regulating FOXP3 (16). Despite relevant strong supporting evidences, the role of miR-31 in endometriosis has not yet been studied.

On the other hand, miR-145 is shown to be a regulator of endothelial cell function and increase cell proliferation and invasiveness in tumors stem cells (17-22). Wang et al. (23) have found that in the serum of patients with endometriosis, miR-145 is down-regulated using circulating miRNA array in comparison to the control women. Therefore, their results have suggested that microRNAmay be a potential biomarker of endometriosis. In addition, miR-145 down-regulates posttranscriptionally the pluripotency factor SOX2 and stemness-associated Musashi RNA binding protein 2 (MSI2). 

It seems that miR-31 with its wide and complex functions in apoptosis, differentiation, proliferation and invasiveness, as well as miR-145 with its proved influence on local invasiveness, proliferation and stemness of endometriotic cells may be involved in the pathogenesis of endometriosis. Considering this hypothesis, we tried to analyze the plasma level of these two miRNAs in patients with endometriosis to have a clear understanding of the pathogenesis of endometriosis and find potential functional biomarkers.

## Materials and Methods

In this case-control study approved by the Tarbiat Modares University Ethics Committee, based on revised American Society of Reproductive Medicine (rASRM) guidelines (24), we recruited 34 patients with histologically proven endometriosis stage 3 or 4 (moderate or severe forms, respectively) and 21 patients with stage 1 or 2 (minimal and mild forms, respectively). There was also a control group including 23 endometriosis-free patients who underwent laparoscopic examination for other indications than infertility, like prolapsed uterus, ovarian cyst, or urinary incontinence. Exclusion criteria of the control group were presence of other endometrial pathologies like myoma or fibroma. Exclusion criteria of the patients groups were the presence of signs and symptoms of endometriosis, like infertility and dysmenorrhea. Exclusion criteria for both patient and control groups were presence of systematic inflammation diseases and infections, ongoing pregnancy, history of pregnancy in the last 3 months, cancer, and other major systemic diseases. The mean age of patients was 28 (23-34) years. All subjects were enrolled in this study after a standard genetic counseling and signing an informed consent form.

To investigate the circulating miRNAs in plasma from patients and controls, 5 ml peripheral blood samples were collected in heparinized tubes before laparoscopy. Then, tubes were centrifuged at 1900 rpm for 10 minutes at room temperature. Plasma was collected in a sterile 1.5-ml microcentrifuge tubes RNase free and cryopreserved at -80°C. To evaluate the expression of hsa-mir-145 and hsa-mir-31 in endometriosis, miRNAs were isolated from samples using miRCURY RNA Isolation Kits-Biofluids (Exiqon, Denmark) according to manufacturers’ instructions. miRNAs were eluted in 50µl of nuclease free water. For first-strand cDNA synthesis reaction, miRCURY LNA™ Universal RT miRNA polymerase chain reaction (PCR), polyadenylation and cDNA synthesis kit (Exiqon, Denmark) were used. Briefly, each reverse transcription reaction consisted of 2 µl 5X reaction buffer, 1 µl enzyme mix, 0.5 µl synthetic RNA spike-ins, 4.5 µl nuclease free water, and a final concentration of 100 ng/µl of total RNA. RT reaction was performed using an Applied BiosystemsVeriti™ thermal cycler (Life Technologies, USA) at 42°C for 10 minutes, followed by heat-inactivation at 95°C for 5 minutes, and stored at 4°C. Real-time PCR was carried out using an Applied Biosystems StepOne Real-Time PCR System (Life Technologies, USA). For quantitative PCR (qPCR), 10 µl PCR reaction mixture was prepared using ExiLENT SYBR® Green PCR master mix (Exiqon, Denmark) as recommended by manufacturer. Briefly, 5 µl of 2XSYBR Green master mix, 4 µl of diluted cDNA (1:4) and 1 µl of LNA primer mix were added. Then, has-mir-103-3p was used as the endogenous control. qPCR was performed at 95°C for 10 minutes for polymerase activation followed by 45 cycles of 95°C for 10 seconds and 60°C for 1 minutes. Finally, melting curve analysis was performed based of the dissociation characteristics of double stranded DNA during cycles with increasing denaturing temperature.

## Statistical analysis

All results were expressed as the mean ± SE. Statistical analysis was performed using GraphPad Prism 5 software (GraphPad, USA). One-way analysis of variance (ANOVA) was applied for comparison of the differences between groups. REST Software was also used to calculate relative quantity (RQ) of the expressions using Pffafl formula.

## Results

Patients presented with infertility and dysmenorrhea in 38 and 29%, respectively. 

**Fig.1:**
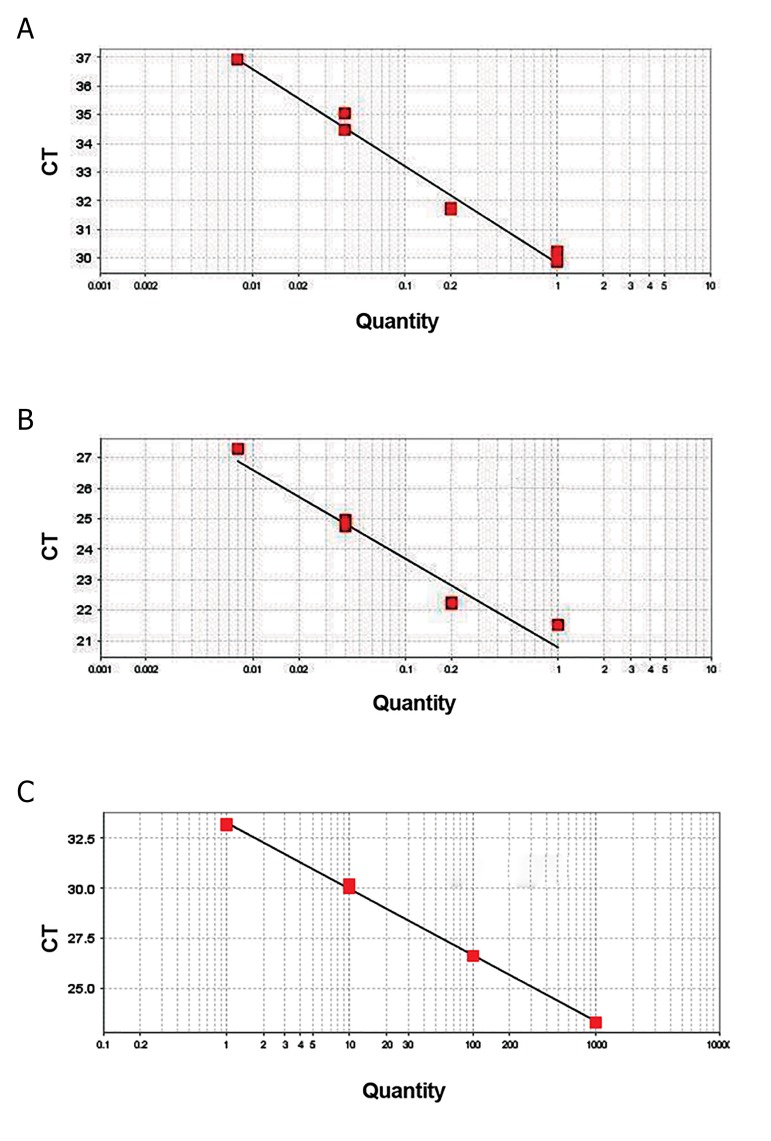
Standard curves. A. miR-31, B. miR-145, and C. miR-103-3p. Efficiencies of amplifications are 97.3 (R^2^=0.98), 101.8 (R^2^=0.974) and 100.958 (R^2^=0.999), respectively. R^2^; The coefficient of correlation and Ct; Cycle threshold.

**Fig.2:**
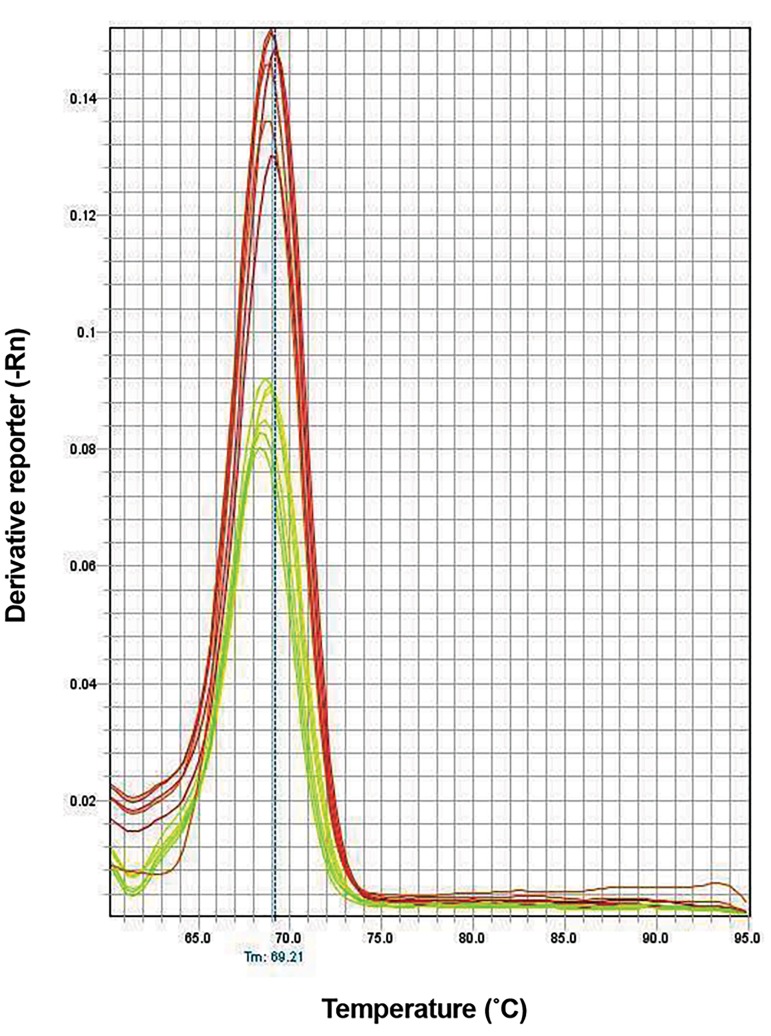
Melting curves of quantitative polymerase chain reactions (qPCR) of miR-31 and miR145. There is no nonspecific or significant dimmer primer peak confirming the specificity of reactions.

**Fig.3:**
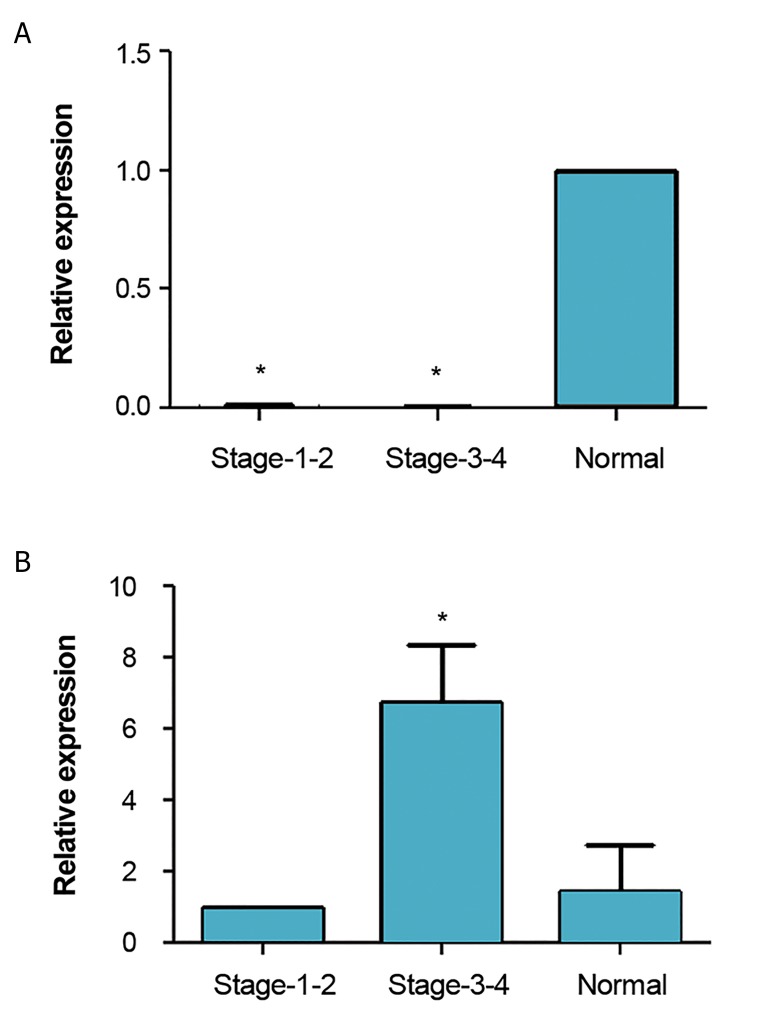
Expression analysis of miR-31 and miR-145 in patients with endometriosisstage 1 or 2 and stage 3 or 4. Relative expressions of different groups are shownin comparison with normal controls. A. The relative expression of miR-31 in stages1 or 2 and stages 3 or 4 compared with Normal and B. The relative expression ofmiR-145 in stages 1 or 2 and stages 3 or 4 compared with normal. In stage 1 or2, the expression level of miR-145 was increased by 6.7-fold (P<0.05), whereas theexpression level of miR-31 was decreased [relative quantity (RQ)<0.01, P<0.05)]. Instage 3 or 4, the expression level of miR-145 was increased by 1.4-fold, suggestingthis change did not reach to a statistical significance level. The expression level of miR-31 was decreased as compared to normal controls (RQ<0.01, P<0.05).

To explore miRNAs profiling from plasma samples, the expression levels of miR-145 and miR-31 were evaluated by qPCR in endometriosis plasma as compared to the control group. The efficiency of qPCR reactions for miR-31 and miR-145 were 97.3 and 101.8, respectively (Fig .1). Melting curves showed no nonspecific or primer dimer peaks (Fig .2). We showed that the expression levels of miR-31 in stage 3 or 4 and stage 1 or 2 were significantly down-regulated (less than 0.01-fold, P<0.05), whereas the expression level of miR-145 was up-regulated in endometriosis women in stage 1 or 2. The expression level of miR-145 in stage 3 or 4 in the patient group was up-regulated more than 1.4-fold, indicating that it did not reach to a significance level. miR-145 was significantly up-regulated in stage 1 or 2 more than 6.7(P<0.05, Fig .3).

## Discussion

Endometriosis is a hormone dependent inflammatory disease, in which the role of pure genetic variations is minor, while environmental and epigenetic factors are mainly involved. Many cellular functions like differentiation, proliferation, mitochondria, reactive oxygen species (ROS) production, invasion and decidualization are deregulated in this disease. miRNAs, including miR-31 and miR-145, regulate many of these biological processes in different pathologies, especially in cancers. Clinical signs and symptoms are insufficient to help an early diagnosis Therefore, there is an urgent need to find biomarkers for early selection of patients with high risk of endometriosis and confirmatory diagnosis by laparoscopy and pathological examination. 

We found that in patients with stage 3 or 4 endometriosis, miR-31 was down-regulated as compared to normal women. In addition, our results demonstrated down- regulation of miR-31 and significant up-regulation of miR-145 in patients with stage 1 or 2 endometriosis. Our findings also indicated that although several miRNAs were deregulated in endometriosis (25), miR-31 and miR145 may be considered as potential biomarkers for noninvasive diagnosis of patients with endometriosis. 

Infertility in patients with endometriosis is due to mechanical as well as functional abnormalities of endometrium. Decidualization defect and abnormal receptivity are the main mechanisms that can explain infertility in endometriosis (5). In concordance, our findings showed that miR-31 as one of the factors influencing decidualization was down-regulated in patients with endometriosis in all stages as compared to endometrium of normal controls. miR-31 is a highly conserved miRNA in evolution that acts as a vital factor in embryonic implantation and development, as well as immune system balance. Deregulation of miR-31 is correlated to some cancers and autoimmune diseases partly by epigenetic modifications, like methylation and acetylation (14). Although many biomarkers in plasma are not functional and as surrogate biomarkers show no role in pathology, some others are involved in controlling the pathogenic processes (25). Due to the regulatory role of miR-31on biological and vital processes in endometriosis, its down-regulation might be an underlying epigenetic mechanism in the pathogenesis. 

Cosar et al. (26) have showed that miR-145 is up- regulated 10-fold higher in patients with endometriosis. However, we found that an increase in miR-145 is mostly in stage 1 or 2, whereas there was a relative decrease in the expression of miR-145 in stage 3 or 4. Adammek et al. (27) have indicated that miR-145 inhibits proliferation, while its over-expression can inhibit proliferation rates up to 45%. In addition, invasiveness was decreased by 80%. A relative decrease in the expression of miR-145 in our patients with moderate to severe endometriosis might promote an increase in proliferation that was seen in more advanced stages of the disease. Although the pathogenesis of endometriosis is unknown, it is believed that endometriosis is a disease of stem cells. Stemness has been studied in endometriosis. There is an overall increase in expression of genes involved in stemness in ectopic as compared to eutopic tissues of patients with endometriosis (28). More specifically, the genes *UTF1, TCL1* and *ZFP42* show a trend for higher expression in endometriosis than in normal endometrium. However, expressions of other genes involved in stemness are not significantly different between endometriosis and normal endometrium. miR-31 is directly correlated to stemness in some cancers. Its expression increases the expression of Nanog/Sox2/oct4 in cancers (29). On the other hand, stemness seems to be de-regulated in eutopic endometrium of patients with endometriosis as compared to normal endometrium. Overexpression of miR-200b which is down-regulated in endometriosis can increase stemness-associated side population phenotype (13). 

miR-145 is also a regulator of stemness. Down- regulation of miR-145 in patients with stage 3 or 4 as compared with patients in stage 1 or 2 indicates the roles of miR-145 in down-regulation of the pluripotency factors and MSI2. A decrease in miR-145 relative expression in more advanced stages might be the underlying cause of increased invasiveness and proliferation, as well as increased expression of stemness related genes, which are found to be up-regulated in endometriosis (13).

Relative down-regulation of miR-145 may promote the progression of the disease from milder stages to more severe stages. In other words, increased expression of miR-145 may inhibit proliferation and promote differentiation (30) in stage 1 or 2, while the disease is still mild. However, when the disease progress to more severe clinical forms, stage 3 or 4, the expression level of miR-145 decreases. It has been shown that increased expression level of miR-145 is involved in infertility and repeated implantation failure (31), which is also the common features of endometriosis.

It is noteworthy that migration and invasion are controlled by miR-31 in cancer cells. Over-expression of miR-31 inhibits MDA-MB-231 cell migration and invasion, while down-regulation of miR-31 promotes MCF-7 cell migration and invasion (32). In addition to invasion inhibition, miR-31 is a proapoptotic agent. *Histone deacetylase inhibitors* (HDACIs) can induce apoptosis through miR-31 induction (33). Endometriosis consists primarily of stromal cells with low rate of apoptosis, little differentiation and more invasiveness (34). Nasu et al. (35) have showed that in endometriosis, there is a resistance to apoptosis. Lack of normal immune responses to endometrial cells, in addition to a decrease in apoptosis, facilitate proliferation and the implantation of ectopic endometrial tissues (36). A decrease in the expression of miR-31 may induce resistance in apoptosis and increase proliferation found in endometriosis. miR31 that is increased during the window of implantation is known as a potential biomarker of optimum receptivity (37). Our findings showed that in the plasma of patients with endometriosis, there is a decrease of miR31 expression level, suggesting that it was a sign of lower receptivity found in endometriosis in previous studies (5, 38). Increased production of estrogens and prostaglandins, as well as progesterone resistance are correlated to the pathogenesis of endometriosis in eutopic tissues of patients with endometriosis as compared to women without endometriosis (39). Progesterone as a differentiating agent is necessary in implantation. Decidualization, which is defected in endometriosis, is under the control of progesterone. A significant decrease in miR-31 that is present in patients with endometriosis could be the underlying cause. As shown in cancers, miRNA has a p53 related anti proliferative activity (40), and p53 has a balancing role between differentiation and proliferation (41). 

Although the pathways are still unclear in endometriosis, recent findings have strongly supported an epigenetic role in the underlying pathology of this disease, especially in miRNAs. Mostly, several miRNAs are important in the pathogenesis and treatment of endometriosis. Since miRNAs expressions are highly specific in tissues and tumors, they may be the potential biomarkers of early diagnosis for the new treatment strategies.

## Conclusion

Overall, we found that miR-31 was under-expressed in patients with endometriosis, while miR-145 is over-expressed in stage 1 or 2 and relatively under-expressed in more severe forms of the disease. It means that deregulated expression of miR-31 may explain the dysfunctions related to this disease. Furthermore, our findings indicated that expression analysis of these miRNAs in the plasma may serve as potential biomarkers in noninvasive diagnosis of endometriosis, while their deregulated expression provides an understanding of the mechanism of pathogenesis of endometriosis.
